# Supplementary material for: Diagnostic Blood Biomarkers in Alzheimer’s Disease
Source: Biomedicines. 2022 Jan 13;10(1):169. doi: 10.3390/biomedicines10010169 (PMC8773964; doi:10.3390/biomedicines10010169)
Supplement: Supplementary file 1 [file biomedicines-10-00169-s001.zip › biomedicines-1528986-supplementary.pdf]

Supplementary Table S1. Participant Demographics

Characteristics	Total	CN	aMCI	AD Dementia	*p*-value
n	136	51	54	31	
Age, mean (SD), y	136	71.5 (5.4)	71.4 (8.4)	65.2 (8.7)	< 0.001
Education, mean (SD), y	132	10.1 (4.9)	9.9 (5.0)	6.4 (3.5)	0.001
Female sex, No. (%)	136	27 (52.9)	22 (40.7)	21 (67.7)	0.055
K-MMSE score, points	131	27.0 (2.2)	25.2 (2.9)	18.9 (4.3)	< 0.001
APOE ε4 carrier, No. (%)	133	19 (37.3)	28 (51.8)	24 (77.4)	0.004
CSF biomarkers, mean (SD), pg/mL					
NFL concentrations, pg/mL	136	806.1 (380.5)	851.8 (376.5)	970.4 (360.9)	0.080
$A\beta_{1-42}$ concentrations, pg/mL	136	830.9 (336.2)	666.2 (279.6)	399.1 (135.1)	< 0.001
t-Tau concentrations, pg/mL	136	260.8 (107.2)	366.2 (213.2)	522.8 (217.1)	< 0.001
p-Tau_{181} concentrations, pg/mL	136	45.2 (15.6)	58.5 (27.0)	74.4 (27.0)	< 0.001
Plasma biomarkers, mean (SD), pg/mL					
NFL concentrations, pg/mL	136	18.6 (6.5)	20.7 (9.4)	21.8 (6.6)	0.050
$A\beta_{1-42}$ concentrations, pg/mL	136	11.5 (3.7)	10.7 (3.3)	8.2 (2.4)	< 0.001
Combination biomarkers, ratio					
CSF NFL/$A\beta_{1-42}$ ratio	136	1.25 (0.98)	1.66 (1.24)	2.62 (1.18)	< 0.001
Plasma NFL/$A\beta_{1-42}$ ratio	136	1.89 (1.10)	2.05 (1.02)	2.92 (1.20)	< 0.001
Neuroimaging data					
Aβ− PET (SUVR score)	135	1.1061 (0.15)	1.1710 (0.23)	1.3886 (0.11)	< 0.001
Hippocampal volume/ICV	134	0.0028 (0.00032)	0.0025 (0.00040)	0.0021 (0.00038)	< 0.001
Entorhinal cortex (mm)	134	3.3826 (0.30533)	3.1671 (0.43704)	2.9403 (0.46155)	< 0.001

Data are presented as means (SD, standard deviation), numbers, or % unless indicated otherwise. Abbreviations: K-MMSE, Korean Mini-Mental State Examination; ICV, intracranial volume; SUVR, standardized uptake value ratio; CSF, cerebrospinal fluid; $A\beta_{1-42}$, amyloid $beta_{1-42}$; t-Tau, total Tau protein, p-Tau, phosphorylated Tau protein; NFL, neurofilament light chain; CN, cognitively normal; aMCI, amnestic mild cognitive impairment; AD, Alzheimer's disease. Differences were analyzed using ANCOVA adjusted for sex and age.
